# Attention-Deficit/Hyperactivity Disorder (ADHD) in Adulthood: Concordance and Differences between Self- and Informant Perspectives on Symptoms and Functional Impairment

**DOI:** 10.1371/journal.pone.0141342

**Published:** 2015-11-03

**Authors:** Beatrice Mörstedt, Salvatore Corbisiero, Hannes Bitto, Rolf-Dieter Stieglitz

**Affiliations:** 1 Department of Psychology, Div. of Clinical Psychology & Psychiatry, University of Basel, Basel, Switzerland; 2 University of Basel Psychiatric Clinics, Basel, Switzerland; University of Florida, UNITED STATES

## Abstract

Attention-deficit/hyperactivity disorder (ADHD) is a severe mental illness, associated with major impairment and a high comorbidity rate. Particularly undiagnosed ADHD in adulthood has serious consequences. Thus, a valid diagnosis is important. In adulthood, the diagnostic process for ADHD is complicated: symptoms may overlap with comorbid disorders, and the onset and progression of the disorder must be reconstructed retrospectively. Guidelines for the diagnostic process recommend the inclusion of additional informant ratings. Research into the relation between self- and informant ratings shows extremely heterogeneous results. The levels of agreement range from low to high. The focus of this study is the concordance and differences between self- and informant ratings on ADHD symptoms and impairments. In this regard, two possible influencing factors (gender and relationship type) are also examined. 114 people participated in this study, 77 with an ADHD diagnosis and 37 without a diagnosis. For all participants, either parents or partners also rated ADHD symptoms and impairments. Small to moderate concordance was found between self- and informant ratings, with females being slightly more concordant than males, particularly for ratings of problems with self-concept. Examination of the consistency within a particular perspective showed that people with ADHD seemed to be unaware of the causal relation between ADHD symptoms and their impairments. A close investigation found almost no influence of gender and relationship type on differences within perspectives. Based on these results, the implications for the diagnostic process are that additional informant information is clearly necessary and helpful.

## Introduction

Attention-deficit/hyperactivity disorder (ADHD) is a severe developmental disorder, appearing in childhood and frequently persisting into adulthood [1, 2]. In adults, the prevalence rate worldwide is between 1% and 7% [3]. Often people also suffer from additional comorbid disorders such as mood disorders, anxiety disorders, substance abuse and personality disorders [4–7].

ADHD is characterized by three core symptoms—*inattention*, *hyperactivity*, and *impulsivity*—and additional symptoms such as *emotional symptoms* [5, 8]. These symptoms, along with deficits in soft skills (e.g. in social communication), lead to severe functional impairment in daily life [9, 10]. People with ADHD report long-term problems in education, at the work place, in family and social life, with leisure activities, and with organization [6, 9, 11–13]. Functional impairment often persists or even increases with age, especially with untreated ADHD [4–6]. Additionally, the disorder has consequences for the social environment of a patient, where notably family functioning is lower within families with members suffering from ADHD [14].

Concerning the wide range of functional impairments and the high rate of comorbidity, an early and valid diagnosis of ADHD is important [4, 15]. Particularly the diagnostic process in adulthood seems to present difficulties: symptoms of ADHD are more heterogeneous than in childhood and can overlap with comorbid disorders [5, 16–18]. To address these problems, specific diagnostic measures for adults have been developed in recent years [19–21] and guidelines for a precise diagnosis established [22–23]. There is evidence that people with ADHD have limited abilities in the areas of self-reflection and self-evaluation [21, 24–25]. Accordingly, their statements might not be valid and can distract from the real problems. Thus, guidelines include a detailed investigation of former and current symptoms by a clinician with inclusion of self- and informant ratings. However, the benefit of informant perspectives for the diagnostic process is under discussion in current research [26–29]. Interobserver agreement seems to be a major limitation here. There are a number of studies showing only low to medium concordance between self- and informant perspectives [27, 29–31]. Nevertheless, some studies found moderate and even high correlations [26, 32, 33].

In many studies, people with ADHD retrospectively rate symptoms in childhood lower than their relatives do [1, 29]. In contrast, they rate their current symptoms in adulthood higher and are more concerned about impairments [1, 28, 30, 31]. In line with these findings, Eakin et al. [34] found that spouses of people with ADHD reported fewer impairments to their married lives than their ADHD partners. In some ratings, they went as far as not to differ from spouses of people without ADHD. Nevertheless, there are also contrary findings: in some studies, people with ADHD estimated the severity of their own symptoms lower than their relatives did [35–37].

Differences in perspectives between people with ADHD and their relatives are rarely discussed in literature. So far, no consensus on the validity of the different perspectives has been reached. There is a lack of objective criteria to evaluate the statements, so it cannot be determined with any certainty which ratings are more precise. Nevertheless, there are some indications as to which perspectives are more valid. In retrospective ratings of childhood symptoms, statements of parents highly correlate with daily impairments in childhood and childhood diagnosis of ADHD, and parents were also better at estimating the time of onset of the disorder [1, 29]. For current ratings in adulthood, results are inconsistent: while some studies show good introspection abilities in people with ADHD [30, 31], others indicate that they have little insight into their problems [36, 37].

These different findings are discussed by various researchers. Some tried to find explanations for the higher symptom self-ratings: one possibility could be an overestimation of the minor executive power limitation found in ADHD [31]. Another explanation is the low self-esteem of people with ADHD and their negative views of themselves [9, 21, 38, 39]. Lower symptom self-ratings found in ADHD patients may be explained by a positive illusory bias: an unawareness of their own symptoms and impairments [40]. Particularly children appear to be unaware of their problems [37, 41, 42]. In adults, this bias was also found, but seems to be less common [26, 36].

Some studies address factors influencing the concordance between the different perspectives. These factors include age, gender, ADHD diagnosis, and the identity of the informants (type of relationship they have with the ADHD person). Zucker et al. [29], for example, only found evidence of a gender influence on ratings of former symptoms in childhood: females rated the severity of their former symptoms higher and more concordant with their relatives. Other studies also found some evidence for the influence of gender: spouses rated their marriages worse if the female partner had ADHD [43, 44]. So far, however, the number of studies investigating influencing factors remains limited.

A deeper investigation of the concordance between perspectives seems necessary and might help to examine their usefulness for diagnostic purposes and therapy. A precise diagnosis is important for the effective treatment of ADHD [4]. Perspectives on symptoms and on functional impairment will be more closely assessed in this study. The following topics will be addressed: (1) internal consistency of the different subscales for self- and informant ratings of ADHD symptoms and functional impairments, (2) the concordance of self-, informant and clinical ADHD diagnosis, (3) the consistency of symptoms and associated impairment within a perspective, (4) examination of the differences and concordance between the self- and the informant perspective, and (5) examination of possible factors influencing the self- and informant ratings. As mentioned above, little is known about the factors influencing the extent of differences in perspectives. Following current research, we considered gender and relationship type as such factors [29]. We assumed that: (a) females are more concordant than males; (b) perspectives between ADHD patients and their partners are more similar than those with parents.

## Method

### Procedures

Data of patients of the ADHD Special Consultations Unit of the Outpatient Department of the University of Basel Psychiatric Clinics, collected between July 2013 and December 2014, were used for this study. The collected routine data was retrospectively used to investigate the research questions of this study. According to the standard approach of the University of Basel Psychiatric Clinics for routine clinical data, informed consent was given verbal, that data may be used for research. Data were collected in anonymous form in a clinical intern data base. None of the authors had contact with the participants and the data was entered anonymized by researcher assistants of the research group. This procedure was approved before the start of the study by the ethics committee in Basel (“*Ethikkommission Nordwest- und Zentralschweiz* (EKNZ”). The EKNZ approved the entire study for the clarification of our research questions with the use of routine data of the special consultations unit.

During the course of several hours, participants were examined for ADHD. In the process, demographic and amnestic information was collected. Former and actual psychiatric disorders and behavior problems were also investigated. Symptomatology of ADHD and comorbidity was gathered with semi-structured interviews (according to the *Adult Interview* of Barkley and Murphy [45]), structured diagnostic interviews and self-, informant, and investigator ratings. Diagnosis was given based on the clinical judgment of two trained psychologists. The procedure conformed to general standards for clinical diagnostics [guidelines: 22].

Diagnoses were made based on DSM-5 [46], according to which at least five symptoms of *inattention* and/or five symptoms of *hyperactivity/impulsivity* must coexist in adulthood. Important components of the process were the *Conners Adult Rating Scales* (CAARS; [19]), in self- and other-forms, and the *Wender-Reimherr Adult Attention Deficits Disorders Scale* (WRAADDS; [21]). Functional impairment was examined with the functional impairment scale (*Barkley Functional Impairment Scale for Adults*; [47]). Former symptoms in childhood were assessed with the *Adult Interview* [45] and the short version of the *Wender Utah Rating Scale* (WURS-k, [48]). Diagnosis was given if participants had current symptoms and also reported former symptoms prior to the age of 12. To check current symptoms, a combination of the results of the clinical interview and the rating scales was used. A diagnosis was given when the answers of the clinical interview indicated an adult ADHD and cut-off-values for the symptoms of most rating scales were fulfilled. In line with ADHD diagnostic guidelines [22–23], the following exclusion criteria were used: estimated Intellectual Quotient IQ < 85, schizophrenia or other psychotic disorders, current or most recent (in the last 3 months) manic episode, severe major depressive disorder, acute stress disorder, substance intoxication and withdrawal (substance dependence was not excluded).

### Participants

This study includes a clinical sample which was routinely diagnosed in the ADHD Special Consultations Unit. A total of 114 persons participated in the study. Of these, 77 were men (67.5%) and 37 women (32.5%), the mean age was 32.19 years (*SD* = 10.82) with an age range from 18 to 75 years. In our sample, 77 participants received an ADHD diagnosis, while 37 did not (ADHD and Non-ADHD group). Demographic characteristics for these two groups are the following: In the Non-ADHD group 24 participants were male (64.9%) and 13 were female (35.1%), while in the ADHD group 53 participants were male (68.8%) and 24 were female (31.2%). The group differences for gender were not significant (*t*(112) = .42; *n*.*s*.). Both groups did neither differ significantly in their mean age (Non-ADHD group *M* = 31.54, *SD* = 13.41; ADHD group *M* = 32.52, *SD* = 9.38; *t*(112) = .45, *n*.*s*.), nor their education: In the Non-ADHD group 21 participants (56.8%) finished secondary school and 16 (43.2%) finished grammar school, while in the ADHD group 50 participants (64.9%) finished secondary school and 27 (35.1%) finished grammar school (*t*(112) = 1.42, *n*.*s*.). There were also no significant differences for relationship status, number of children, and the informant identity between both groups.

No participant of the ADHD group was receiving specific pharmacological or psychological therapy for ADHD during the time of the assessment. There were no significant group differences in gender or age.

For 65 (57.0%) participants, also partners rated symptoms and impairment, while for the rest (43.0%) parents did.

In the ADHD group, 47 people (61.0%) had a current comorbid disorder such as a mood disorder, anxiety disorder or a substance use disorder. People in the ADHD group had significantly more former depressions (*t*(107) = 2.40, *p* < .05), more anxiety problems (*t*(105) = 3.32, *p <* .01), more thoughts about suicide (*t*(112) = 2.87, *p* < .01), more alcohol abuse (*t*(109) = 3.19, *p* < .01), and more substance abuse (*t*(111) = 2.79, *p* < .01).

### Measures

Only the instruments used for this study are described in more detail here.

*Conners Adult Rating Scales (*CAARS; [19], German version: [49, 50]). The CAARS is a questionnaire consisting of different versions, each of which was developed to measure the presence and severity of ADHD symptoms in adults. For our study, we used the long-version self-report scale (CAARS-S: L) and the long-version observer rating scale (CAARS-O: L). Both of these address the same behaviors and contain identical scales, subscales, and indices. ADHD symptoms are examined through 66 items for both versions. Christiansen et al. [49, 50] validated the German version of the questionnaire within two studies. Similar as Conners et al. [19], they found a moderate (Cronbach`s α >.70) to good internal consistency (Cronbach`s α >.80) [49, 50]. They also reported a high internal reliability for the CAARS-S, with *r* = .74 to .93, which indicates a high stability of the subscales. The CAARS:O also showed a moderate to high inter-rater reliability with *r* = .64 to .85. To assess the diagnosis based on self- and informant ratings, the *DSM-IV total ADHD symptoms scale* was dichotomized. The cut-off point for receiving a diagnosis, with respect to norms of gender and age, was set to a t-value of 60 according to [19]. For continuous calculations the *ADHD index* and its subscales of *inattention/memory problems*, *hyperactivity/restlessness*, *impulsivity/emotional lability* and *problems with self-concept* were utilized.

*Barkley Functional Impairment Scale for Adults* (BFIS; [46]). The BFIS is a questionnaire assessing 15 major domains of psychosocial functioning in adults. Two forms are available: self- and other-report. With a Cronbach`s alpha of .97, Barkley [46] found a good internal consistency of the total score of impairment for the self-report (*mean impairment score*). The interobserver agreement for each item was between .44 and .77. Till now, there is no published German version of this questionnaire available. Therefor we used a translation, made by our research group. Because of the small participants’ number, a validation of the questionnaire based on our data was not advisable. For the assessment of functional impairment in our study, four domains of impairment were formed out of cluster analysis results: *family life*, *social life*, *work*, and *organization*. Cronbach`s alphas for the *mean impairment score* and the subscales can be found in the results section.

*Adult Interview* (AI; [47]). This is an interview focusing on ADHD pathology, comorbidity, functional impairment and pervasiveness. For this study a German translation from our research group was used. To our knowledge, there is no published German version available. There are no psychometric properties and normative data available for the interview yet. The interview was used to assess comorbidity.

### Data analysis

Internal consistency was assessed with Cronbach`s alpha. Values were calculated for all subscales of ADHD symptoms and all domains of impairments for self- and informant ratings.

Concordance between clinical diagnosis and the diagnosis based on self- and informant statements was examined with Spearman correlations and cross tabulations. Thereby the percent of similarity between the diagnosis (between clinical diagnoses and diagnosis based on self-statements; between clinical diagnosis and diagnosis based on informant statements, and between diagnosis based on self-statements and diagnosis based on informant statements) were calculated.

Regression analyses with the scales of *mean impairment score* (BFIS) and *ADHD index* (CAARS) were used to test for consistency of symptoms and associated impairment within self- and informant perspectives. The ratio of explained variance should be an indicator for consistency of the perspectives.

General differences between self- and informant perspectives were examined for both groups separately through t-tests. For differences, also effect sizes (Cohen`s d) were computed.

In-depth analysis of the relationship between self- and informant perspectives in ADHD was assessed with the ADHD group only: Spearman correlations for intra-class concordance of self- and informant ratings and Multivariate Analysis of Covariance (MANCOVA) to test the influence of gender and relationship type were used. Via Fisher z-transformations the intra-class correlations were normalized and t-tests conducted. Restricted sample size demanded separate MANCOVAs for the influence of gender and for the relationship type, controlled for comorbidity. Direction, significance, and effect size for the different subscales are reported.

## Results

### Internal consistency of self- and informant ratings

Reliability of the *ADHD index*, the five ADHD symptoms, the functional impairment domains, and the *mean impairment score* were assessed with Cronbach`s alpha. The following values are the results for (a) self- and (b) informant ratings. For the *ADHD index* scale, Cronbach`s alpha (a) was .93 and (b) .93. Results for separate ADHD symptoms were as follows: for *inattention/memory problems* Cronbach`s alpha (a) was .87 and (b) .90, for *hyperactivity/restlessness* Cronbach`s alpha (a) was .89 and (b) .90, for *impulsivity* Cronbach`s alpha (a) was .80 and (b) .83, for *emotional lability* Cronbach`s alpha (a) was .85 and (b) .84, and for *problems with self-concept* Cronbach`s alpha (a) was .87 and (b) .84. The *mean impairment score* showed Cronbach`s alpha of (a) .84 and (b) .93, while for the different impairment domains the following results were found: for *family life* Cronbach`s alpha (a) was .79 and (b) .80, for *social life* Cronbach`s alpha (a) was .81 and (b) .81, for *work* Cronbach`s alpha (a) was .57 and (b) .71, and for *organization* Cronbach`s alpha (a) was .82 and (b) .82. Taken together, internal consistency showed good to excellent values for the total scales, the subscales of ADHD symptoms, and almost all separate domains of impairment for both ratings. An exception was *work*, which showed low values for self-ratings but acceptable values for informant ratings.

### Concordance of self-, informant, and clinical ADHD diagnosis

A moderate correlation (*r* = .53) and a general similarity of 80% (44% for no diagnosis and 97% for diagnosis) were revealed for an ADHD diagnosis based on clinical diagnosis and self-ratings. For informant ratings and clinical diagnosis correlation was small (*r* = .27) and a general similarity of 68% (51% for no diagnosis and 76% for diagnosis) was found. The agreement between self- and informant diagnosis was also small: *r* = .22 and general similarity was 70% (56% for no diagnosis and 72% for diagnosis).

### Consistency of symptoms and associated impairment within a perspective

To examine the consistency of a perspective, hierarchical multiple regression analyses were used. Thus, the prediction of ratings for impairment through symptom ratings was tested separately for the participants and their relatives in both groups (ADHD and Non-ADHD group). For this purpose, the *mean impairment score* of the BFIS and the *ADHD index* scale of the CAARS were used as self- and informant ratings. For the Non-ADHD group both perspectives were consistent: for self- ratings the ADHD symptoms explained 75% of the total variance of the impairment scale (*F*(1, 34) = 99.85, *p* < .001), and for informant ratings the symptom scale explained 52% of the total variance (*F*(1, 34) = 37.21, *p* < .001). Findings for the ADHD group were different: self-ratings of symptoms explained only 16% of the total variance (*F*(1, 74) = 13.80, *p* < .001), while for informant ratings the symptom scale explained still 41% of the total variance (*F*(1, 72) = 50.57, *p* < .001). To rule out comorbid diseases as the basic cause for lack of consistency within the ADHD self-ratings, a subgroup of ADHD patients without comorbidity was retested (*n* = 30). In self-ratings of this group, symptoms explained 7% of the total variance (*F*(1, 28) = 2.09, *n*.*s*.), while for informant ratings the symptom scale explained 41% of the total variance (*F*(1, 26) = 17.77, *p* < .001).

### Differences in perspectives between participants and their relatives

Independent-samples t-tests were used to evaluate ratings of ADHD symptoms and functional impairments between participants and their relatives. Comparisons between both perspectives showed significant differences for ADHD symptoms and general impairment in the ADHD group, while in the Non-ADHD group no significant differences were found. Calculations were made with the *ADHD index* subscale of the CAARS-S:L and CAARS-O:L and the *mean impairment score* of the BFIS, also self- and others-rating form. No significant differences were found in the Non-ADHS group: *ADHS index*: Participants with ADHD (*M* = 1.13, *SD* = 0.44) and informants (*M* = 1.11, *SD* = 0.50; *t*(35) = 0.27, *n*.*s*.; Cohens *d* = .04); *Mean impairment score*: Participants with ADHD (*M* = 3.49, *SD* = 1.69) and informants (*M* = 3.08, *SD* = 1.77; *t*(35) = 1.19, *n*.*s*.; Cohens *d* = .24). In the ADHD group significant group differences were found: *ADHS index*: Participants with ADHD (*M* = 1.73, *SD* = 0.38) and informants (*M* = 1.53, *SD* = 0.47; *t*(75) = 3.91, *p* < .001; Cohens *d* = .47); *Mean impairment score*: Participants with ADHD (*M* = 4.91, *SD* = 1.72) and informants (*M* = 4.30, *SD* = 1.95; *t*(73) = 2.62, *p* < .05; Cohens *d* = .33).

### Concordance between people with ADHD and their relatives

Concordance of the *ADHD index*, ADHD symptom subscales, and the *mean impairment score* were tested with mean Spearman correlations (Table 1). To this end, correlations for the whole ADHD group and separately for women and men were calculated. Most correlations for symptoms were around .30 and .40, indicating small to moderate correlations. Correlations differed between females and males: for some subscales (*hyperactivity*, *problems with self-concept*, *ADHD index*, and *mean impairment score*) women were more concordant with their relatives than men. This difference was only significant for ratings of *problems with self-concept*.

**Table 1:** Mean Spearman correlation of self- and informant ratings and comparison of female and male correlations on ADHD symptoms and general functional impairment.

	*All*	*females*	*males*	*df*	*t-value*	*Cohen`s d*
ADHD symptoms						
Inattention/memory problems	.306***	.302***	.308***	72	.28	.07
Hyperactivity/restlessness	.324***	.392***	.291***	73	1.25	.31
Impulsivity	.380***	.362**	.388***	65	.15	.04
Emotional lability	.381***	.370**	.387***	62	.24	.06
Problems with self-concept	.263***	.499***	.151	60	3.31**	.88
ADHD index	.383***	.445***	.354***	74	1.35	.35
Impairment						
Mean impairment score	.486***	.557***	.452***	70	1.75	.43

*Notes*. CAARS-S:L and CAARS-O:L: ADHD symptoms; BFIS-S and BFIS-F: impairment domains. Only ADHD group, *N* = 78, females: *n* = 24; males: *n* = 53.

** p < .01*;*

*** p < .001.

Effect sizes: > 0.2 small; > 0.5 medium; > 0.8 large.

^1^ Fisher z-transformation applied.

### Gender, relationship type, and differences in perspectives

The gender of the ADHD person and the relationship type (parents vs. partners) were investigated as possible factors influencing the differences in perspectives between people with ADHD and their relatives. For the purpose of these analyses only the ADHD group was used. Two MANCOVAs were calculated: one with perspectives and gender (Tables 2 and 3), the other with perspectives and relationship type as independent variables (Tables 4 and 5). In both analyses comorbidity was controlled.

**Table 2:** Estimated means of the different groups (perspectives and gender).

ADHD Symptom/Impairment	ADHD adult		informant	
	female	male	female	male
	*M (SE)*	*M (SE)*	*M (SE)*	*M (SE)*
Inattention/Memory Problems	1.86 (0.64)	1.74 (0.55)	1.54 (0.62)	1.73 (0.63)
Hyperactivity/Restlessness	1.57 (0.46)	1.78 (0.60)	1.17 (0.52)	1.59 (0.68)
Impulsivity	1.61 (0.63)	1.57 (0.59)	1.17 (0.48)	1.33 (0.74)
Emotional Lability	2.01 (0.64)	1.71 (0.66)	1.60 (0.70)	1.63 (0.74)
Problems with Self-Concept	2.18 (0.56)	1.51 (0.71)	1.85 (0.69)	1.45 (0.75)
Family life	5.05 (2.31)	4.60 (2.16)	4.40 (2.09)	4.50 (2.47)
Social life	4.19 (2.51)	4.38 (2.50)	3.19 (2.56)	3.49 (2.46)
Work	5.34 (2.30)	4.63 (2.24)	4.58 (2.34)	4.14 (2.70)
Organization	5.23 (2.46)	5.33 (2.07)	4.36 (2.18)	4.67 (2.45)

*Notes*. CAARS-S:L and CAARS-O:L: ADHD symptoms; BFIS-S and BFIS-F: impairment domains. Only ADHD group, *N* = 77.

**Table 3:** Effects of the multivariate analyses of covariance (MANCOVA) in the different groups on the ADHD symptoms and impairment.

ADHD Symptom/ Impairment	Main effect perspective		Main effect gender		Interaction perspective* gender	
	*F(2*, *75)*	*η* ^*2*^	*F(2*, *75)*	*η* ^*2*^	*F(2*, *75)*	*η* ^*2*^
Inattention/Memory Problems	0.15	.00	0.24	.00	3.38	.04
Hyperactivity/ Restlessness	14.19***	.16	5.79*	.07	3.73	.05
Impulsivity	6.07*	.08	0.15	.00	1.44	.02
Emotional Lability	1.87	.03	0.26	.00	3.72	.05
Problems with Self-Concept	0.47	.01	10.17**	.12	2.27	.03
Family life	0.32	.01	0.04	.00	0.51	.01
Social life	1.48	.02	0.86	.01	0.01	.00
Work	0.02	.00	1.04	.02	0.04	.00
Organization	0.25	.00	0.34	.01	0.04	.00

*Notes*. CAARS-S:L and CAARS-O:L: ADHD symptoms; BFIS-S and BFIS-F: impairment domains. Only ADHD group, *N* = 78.

* p < .05*;*

** p < .01*;*

*** p < .001.

Results controlled for influence of comorbidity.

**Table 4:** Estimated means of the different groups (perspectives and relationship type).

ADHD Symptom/ Impairment	ADHD adult		informant	
	Compared with parents	Compared with partners	Compared with parents	Compared with partners
	*M (SE)*	*M (SE)*	*M (SE)*	*M (SE)*
Inattention/Memory Problems	1.71 (0.62)	1.81 (0.55)	1.57 (0.64)	1.73 (0.62)
Hyperactivity/Restlessness	1.71 (0.59)	1.71 (0.56)	1.60 (0.64)	1.38 (0.66)
Impulsivity	1.55 (0.51)	1.60 (0.65)	1.15 (0.64)	1.35 (0.69)
Emotional Lability	1.76 (0.67)	1.83 (0.67)	1.61 (0.70)	1.63 (0.74)
Problems with Self-Concept	1.75 (0.59)	1.70 (0.81)	1.54 (0.74)	1.60 (0.77)
Family life	4.14 (2.47)	5.05 (2.02)	4.34 (2.74)	4.53 (2.13)
Social life	3.76 (2.67)	4.62 (2.36)	3.21 (2.63)	3.50 (2.42)
Work	4.83 (2.53)	4.86 (2.15)	3.92 (3.25)	4.46 (2.17)
Organization	5.37 (2.53)	5.26 (2.00)	4.62 (2.78)	4.54 (2.12)

*Notes*. CAARS-S:L and CAARS-O:L: ADHD symptoms; BFIS-S and BFIS-F: impairment domains. Only ADHD group, *N* = 78.

**Table 5:** Effects of the multivariate analyses of covariance (MANCOVA) in the different groups on the ADHD symptoms and impairments.

ADHD Symptom/ Impairment	Main effect perspective		Main effect relationship		Interaction perspective* relationship	
	*F(2*, *75)*	*η* ^*2*^	*F(2*, *75)*	*η* ^*2*^	*F(2*, *75)*	*η* ^*2*^
Inattention/Memory Problems	1.13	.02	0.52	.01	1.15	.02
Hyperactivity/Restlessness	11.07**	.13	0.94	.01	4.70*	.06
Impulsivity	4.77*	.06	1.13	.02	1.26	.02
Emotional Lability	0.54	.01	0.20	.00	0.02	.00
Problems with Self-Concept	0.03	.00	0.40	.01	1.10	.02
Family life	0.78	.01	0.80	.01	0.80	.01
Social life	1.50	.02	0.31	.00	0.62	.01
Work	0.03	.00	0.22	.00	1.16	.02
Organization	0.23	.00	0.19	.00	0.13	.00

*Notes*. CAARS-S:L and CAARS-O:L: ADHD symptoms; BFIS-S and BFIS-F: impairment domains. Only ADHD group, *N* = 78.

* p < .05

** *p* < .01.

Results controlled for influence of comorbidity.

There was a main effect on perspectives found in *hyperactivity/restlessness* and *impulsivity*: people with ADHD themselves rated their symptoms higher than their relatives did.

For gender, a main effect on ratings of *hyperactivity/restlessness* and *problems with self-concept* was found. For *hyperactivity/restlessness* males rated their symptoms significantly higher than females, while for *problems with self-concept* the contrary was found. The biggest effect was found on *problems with self-concept*.

For relationship type, no main effects were found. For *hyperactivity/restlessness*, there was a significant correlation between perspective and relationship type: while participants rated similar symptoms in both groups, parents rated the problems of their adult children higher than partners did.

## Discussion

The main goal of this study is the investigation of the self- and informant ratings on symptoms and impairment in ADHD. For this purpose, concordance and differences between the two perspectives were addressed. While concordance measured the similarity of patterns of both perspectives, with differences the levels of agreement between both perspectives were considered. Additionally, the consistency within a perspective and also influencing factors for differences between ratings were observed.

Examination of the agreement between self-ratings and clinical ADHD diagnosis revealed higher similarity compared to agreement between informant ratings and clinical diagnosis. This is hardly surprising, bearing in mind that judgments of clinicians mostly depend on the statements of patients. Nevertheless, participants rated their symptoms as clinically relevant more often than did their clinicians, which indicates that although clinicians depend on the statements of patients, they interpret this information carefully. Furthermore, this probable overestimation of ADHD symptoms by patients themselves leads to the conclusion that informants may be important for a second perspective within the diagnostic process.

Results about consistency within the perspectives reveal that in the Non-ADHD group severity of symptoms predicted severity of impairment in self- as well as informant rating, while in the ADHD group only the informant ratings achieved this result. Self-ratings in the ADHD group only explained a small amount of the variance in impairment. These results became even more pronounced after excluding the influence of comorbidity: while informant ratings stayed the same, self-ratings became insignificant. In general people with ADHD rated more symptoms and impairments, compared with their informants. But our results suggest that these ratings are very variable: While some participants with ADHD rated higher symptoms, but lower impairments, others rated lower symptoms, but higher impairments. This indicates that people with ADHD seem to be unaware of the connection between their symptoms and associated impairment. Also an unawareness of a causal connection between psychological symptoms and impairments is documented for some other disorders [51], it is notable that in our sample *only* people with ADHD failed to see these connections. In contrast, a review of Gordon et al. [52] showed a general lack of the connection between symptoms and impairments in children with ADHD. Our findings may plead for the assumption that the abilities of adults with ADHD for introspection are limited and their self-view is incoherent. This is in line with previous findings that ADHD patients have little insight into their problems [24, 25, 36, 37]. Accordingly, their perspective may be biased and they may not be reliable informants in respect of their own problems. This outcome underlines the importance of informant ratings for a valid ADHD diagnosis.

Correlations between self- and informant ratings indicate a small to moderate concordance between perspectives, testing the whole ADHD group. The following gender differences in concordance were found: for most ratings females were slightly more concordant than men, which was significant for *problems with self-concepts*. While females showed moderate concordance for this subscale, ratings of males with ADHD hardly correlated with those of their relatives. Higher concordance between females and their relatives corresponds with our hypothesis and findings of former studies [29].

Differences between self- and informant ratings were also examined. General differences in mean scores indicate that people with ADHD estimate their total symptoms and impairments significantly higher than their relatives. Further investigations for single ADHD symptom subscales revealed that these differences were significant for *hyperactivity/restlessness* and *impulsivity*. The modification of these two groups of symptoms with increasing age can be seen as an explanation: both become more internal with increasing age [53–55]. Thus, former external fidgeting turns into inner restlessness and problems with relaxation, while former impulsive decisions and outbursts of temper decrease and people begin to act more sensibly [56]. Consequently, relatives may underestimate these problems, while patients themselves continue to feel impaired. By contrast, *inattention* symptoms do not decline with age and thus may be relatively better recognized in adulthood. Another explanation for these results may be the negative view people have of *inattention* in other people: negative feelings in relationships and social dysfunctioning are more closely connected with *inattention* symptoms than with *hyperactive* behavior [44, 57]. Also, Canu and Carlson [58] reported that *impulsive* and *hyperactive* or *impulsive* behavior is less negative for flirting partners: in contrast to *inattention*, it is perceived more as an interesting personality trait, useful in dating and social relations. However, also an overestimation of symptom severity by people with ADHD themselves cannot be ruled out as explanation for the differences [9, 38, 39]. Robbins [59], for example, assumed that persistent problems with social and family environment lead to low self-esteem and a negative self-view. Accordingly, the higher ratings may be a memory of symptoms in childhood instead of a depiction of actual problems.

For functional impairment, significant differences for the *general impairment score* were found, while the separate ratings of the four domains of impairment (*social life*, *family life*, *work*, and *organization*) did not differ between self and informant. Thus, patients and their relatives generally tell of similar problems in different domains of daily life. Results are in line with former studies, showing high impact of ADHD on daily life [2, 7, 11].

General differences in perspectives on symptoms and impairment were more closely investigated with additional MANCOVAs, with examination of possible influences of gender and relationship type. For gender, we found a main effect on symptom ratings in respect of *hyperactivity* and *problems with self-concept*. For *hyperactivity*, males had significantly higher values, while for *problems with self-concept* females rated higher. An explanation for the higher ratings in men may be the differences in symptoms between females and males: according to former studies, men show more symptoms of *hyperactivity* than women in adulthood (overview: [60]). In contrast, females seem to be more socially disadvantaged by ADHD symptoms through their gender-roles than males [44]. Thus, they may have more problems with social and family life, leading to lower self-esteem and a lower self-concept. Calculations for gender differences in perspectives on impairment yielded no significant results: general means did not differ between females and males. This is in line with the results for concordance for the *mean impairment score*, showing similar moderate correlations for both genders.

Outcomes also revealed that the relationship type seems to be unimportant for general accordance between people with ADHD and their informants. Only for *hyperactivity/ restlessness* were differences found: parents seem to estimate the severity of symptoms of their adult children higher than partners did. The higher ratings by parents may be because they remembered former childhood problems instead of restricting their rating to current symptoms in adulthood only. Another explanation may be personal perception: as already described above, partners may see these symptoms as personality traits they like, while parents see them in terms of clinical relevance. At the beginning, we hypothesized that partners’ views may be more useful because of a closer personal relationship between partners in adulthood. This hypothesis was disproved: both calculations of concordance and of differences showed similar results, which indicates that relationship type bears no significant influence on similarities or differences between perspectives. It is noteworthy that particularly for people under the age of 30 it tended to be parents who provided the informant ratings. Accordingly, depending on the age of the person with ADHD, parents as well as partners are important sources and can and should be used for the diagnostic process.

### Limitations and future directions

For the purpose of this study, two questionnaires in self- and informant report forms were used. This has its strengths and limitations. This procedure enabled perspectives to be compared in detail: the same questions were used for participants themselves and their informants and the same subscales were examined. Other instruments to assess perception of severity of symptoms and impairments in more detail were not included. For future studies, therefore, inclusion of more such measurements may be appropriate.

A second limitation is our sample size. In the Non-ADHD group, only 37 participants and their relatives were tested, while the ADHD group consisted of 78 people. In both groups, others’ perspectives were assessed for either parents *or* partners only. This limits the group sizes. Accordingly, a MANCOVA containing gender and relationship type as influencing factors could not be conducted. For future investigations more participants should be assessed. Also, the inclusion of parents *and* partner ratings for all participants would be meaningful. This procedure would allow a closer comparison of perspectives and enable possible significant differences between partners and parents to be detected.

Finding a valid external criterion for the accuracy of ratings is a considerable problem in studies on self- and informant ratings. For future research, methods such as the ones used by Adler et al. [61] might be interesting: in their longitudinal study, they demonstrated changes in concordance between self- and rater reports within ADHD therapy. They compared former self-ratings of ADHD symptoms and ratings of clinicians with therapy success and later ratings and concluded that rater perspectives were more valid at the beginning. The same design may be useful to investigate the perspectives of people with ADHD and their informants in further investigations.

The last limitation emerges from the assessment of comorbidity. Only a background interview, asking participants for their psychiatric backgrounds, former /actual therapies, and former /actual disorders was used. For further research comorbidity should be evaluate more closely by employing a structured interview for mental disorders. Also the assessment of other neurodevelopmental disorders, like autism, and for seizures should be included. This approach may help to better understand connections between ADHD, perspectives, and comorbidity. Despite these limitations, results show that self- as well as informant ratings should be an important part of the ADHD diagnostic process. Results revealed a low consistency within self-ratings and an overestimation of symptoms in comparison with the clinical diagnosis. Thus, both perspectives are pertinent for clinicians to better understand the individual person with ADHD.

Furthermore, impairments in family and social life indicate that including family members in therapy might be reasonable. Former research showed that medication for ADHD has no effect on social and family impairment [62, 63]. By now, most psychological therapy programs for ADHD do not include modules of family or couple therapy. Nevertheless, systemic therapy tools should be considered as integral elements in therapy, for example couple therapies (e.g., integrative behavioral couples therapy; [64].

### Conclusion

In conclusion, people with ADHD and their relatives are important sources for assessment of symptoms and functional impairment. Nevertheless, there are some significant differences between their respective perspectives of symptoms and impairments. So far, no objective measurement for symptoms and impairments is available, wherefore the level of accuracy of information obtained from patients and other informants remains uncertain. People with ADHD themselves make no causal link between their symptoms and their impairments. The resulting inconsistency within their ratings can be an indication for misjudgment. Accordingly, the additional information informants can give in support of the diagnostic process would appear to be essential.
